# Multiplex gene editing via CRISPR/Cas9 exhibits desirable muscle hypertrophy without detectable off-target effects in sheep

**DOI:** 10.1038/srep32271

**Published:** 2016-08-26

**Authors:** Xiaolong Wang, Yiyuan Niu, Jiankui Zhou, Honghao Yu, Qifang Kou, Anmin Lei, Xiaoe Zhao, Hailong Yan, Bei Cai, Qiaoyan Shen, Shiwei Zhou, Haijing Zhu, Guangxian Zhou, Wenzhi Niu, Jinlian Hua, Yu Jiang, Xingxu Huang, Baohua Ma, Yulin Chen

**Affiliations:** 1 College of Animal Science and Technology, Northwest A&F University, Yangling 712100, China; 2 MOE Key Laboratory of Model Animal for Disease Study, Model Animal Research Center of Nanjing University, National Resource Center for Mutant Mice, Nanjing 210061, China; 3 School of Life Science and Technology, ShanghaiTech University, Shanghai 201210, China; 4 College of Life Science, Yulin University, Yulin 719000, China; 5 Ningxia Tianyuan Sheep Farm, Hongsibu, 751999, China; 6 College of Veterinary Medicine, Northwest A&F University, Yangling 712100, China

## Abstract

The CRISPR/Cas9 system provides a flexible approach for genome engineering of genetic loci. Here, we successfully achieved precise gene targeting in sheep by co-injecting one-cell-stage embryos with Cas9 mRNA and RNA guides targeting three genes (*MSTN*, *ASIP*, and *BCO2*). We carefully examined the sgRNAs:Cas9-mediated targeting effects in injected embryos, somatic tissues, as well as gonads via cloning and sequencing. The targeting efficiencies in these three genes were within the range of 27–33% in generated lambs, and that of simultaneously targeting the three genes was 5.6%, which demonstrated that micro-injection of zygotes is an efficient approach for generating gene-modified sheep. Interestingly, we observed that disruption of the *MSTN* gene resulted in the desired muscle hypertrophy that is characterized by enlarged myofibers, thereby providing the first detailed evidence supporting that gene modifications had occurred at both the genetic and morphological levels. In addition, prescreening for the off-target effect of sgRNAs was performed on fibroblasts before microinjection, to ensure that no detectable off-target mutations from founder animals existed. Our findings suggested that the CRISPR/Cas9 method can be exploited as a powerful tool for livestock improvement by simultaneously targeting multiple genes that are responsible for economically significant traits.

Sheep are an economically important livestock that serve as a resource for various products (e.g., meat, wool, and milk), as well as a significant disease model in biomedical research, including bone healing^1^,^2^, cardiology^3^, and reproduction^4^. Thus, the application of genetic engineering in sheep may potentially accelerate sheep breeding, as well as contribute to the development of better therapeutic approaches for chronic human diseases.

Programmable nucleases, which include zinc finger nucleases (ZFNs) and transcription activator-like effector nucleases (TALENs), enable site-directed engineering of the genome in many cell lines and organisms. Gene-modified sheep were produced using lentiviral vectors^5^ and RNA interference (RNAi)^6^, or through reprogrammable ZFNs^7^ and TALENs^8^, demonstrate the potential of targeting specific genes in sheep. Recently, the clustered regulatory interspaced short palindromic repeat (CRISPR)-associated (Cas)-based RNA guided DNA endonuclease such as the *Streptococcus pyogenes* Cas9 nuclease (CRISPR/Cas9) has enabled rapid genome editing by deleting, adding, activating, or suppressing targeted genes at a very high efficiency and specificity in a wide spectrum of organisms including human cells^9^,^10^, crops^11^,^12^ and large animals (such as in pigs^13^,^14^, goats^15^,^16^, and dogs^17^). Cas9-mediated knockout in sheep have been demonstrated^18^,^19^, opening an avenue for improving sheep breeding by genetic engineering. Nevertheless, whether genetic engineering improves economic traits remains to be clarified. In addition, most economic traits are attributed by multiple genes. Therefore, efficiently targeting multiple loci simultaneously, and the expression of desired traits in sheep remains to be established.

To this end, in the present study, we targeted three functional genes, including the myostatin (*MSTN*), agouti signaling protein (*ASIP*), and beta-carotene oxygenase 2 (*BCO2*), by Cas9/sgRNA, and carefully analyzed the phenotype resulting from the disruption of the *MSTN* gene. The *MSTN* gene is considered as a predominant target choice for genetic engineering because it is a negative regulator of muscle growth in sheep^20^,^21^,^22^. The *ASIP* gene is responsible for coat color patterns in sheep^23^,^24^,^25^,^26^, and a duplicated region of this gene is responsible for the white vs. black coat in sheep^23^. A nonsense mutation (c.196C > T) in the *BCO2* gene is associated with the yellow fat color in sheep^27^. The carcass with yellow fat (also known as yellow fat disease or panniculitis), occasionally observed in sheep leads to metabolic diseases and may sometimes be lethal^28^. These results demonstrate the efficient multiple gene targeting by CRISPR/Cas9, and provide the first detailed evidence of economic trait improvement by gene targeting in sheep.

## Results

### sgRNAs design and validation in sheep fibroblasts and injected zygotes

**Figure 1:**
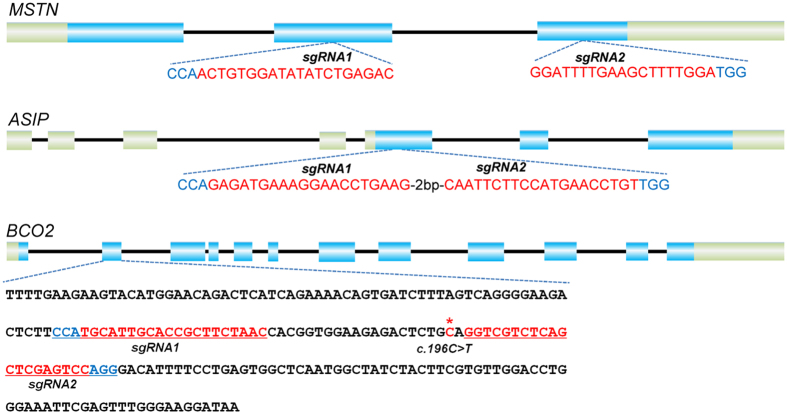
Schematic diagram of genetic structures of *MSTN*, *ASIP* and *BCO2* and their targeting loci of sgRNA:Cas9. sgRNAs targeting sites are highlighted in red, whereas PAM sequences are highlighted in blue. The asterisk indicates the location of the c.196C > T mutation in the *BCO2* gene.

To determine the potential of CRISPR/Cas9 system, and evaluate the efficiency of multiple gene editing simultaneously, three genes (*MSTN*, *ASIP*, and *BCO2*) with known functions in sheep were selected. sgRNAs independently targeting exon 2 and 3 of *MSTN*, exon 5 of *ASIP* and exon 2 of *BCO2* (Fig. 1, Supplementary Table S1) were designed as previously described^9^. Subsequently, the Cas9 and sgRNAs of the three target genes were transcribed *in vitro* by T7 RNA polymerase as previously described^29^. Fibroblasts isolated from Tan sheep were used to validate the activity of these sgRNAs. Genotyping using T7 endonuclease I (T7EI) showed that PCR fragments from genome targeting by sgRNAs were cut into expected bands (Supplementary Fig. S1a,b,d and f), indicating that the CRISPR/Cas9 system can mediate efficient genome editing in sheep fibroblasts. Sanger sequencing further confirmed the existence of different genotypes due to target modifications in sheep fibroblasts (Supplementary Fig. S1c,e and g, Supplementary Table S2).

Based on the observed disruption of *MSTN*, *ASIP*, and *BCO2* in sheep fibroblasts via the CRISPR/Cas9 system, we further investigated its efficiency in developing zygotes. A total of 20 sheep early embryos (one-cell stage) from three donors were surgically collected from naturally mated sheep through the superovulation approach. Approximately 20 ng/μL of Cas9 mRNA and 5 ng/μL of each sgRNA from the *MSTN*, *ASIP*, and *BCO2* genes were pooled and microinjected into 20 sheep embryos (Table 1). After 168 h of culture, genomic DNA was isolated from 20 individual embryos, and screened for the presence of site-specific gene modification by PCR amplification of the region around the targeted site and T7EI cleavage assay (Supplementary Fig. S2a–d). The cleavage was further characterized by Sanger sequencing, which displayed overlapping peaks in the sequencing chromatographs (Supplementary Fig. S2e–h), demonstrating distinguishable indels at the target sites of these three genes (*MSTN*, *ASIP*, and *BCO2*). These data showed that the designed sgRNAs efficiently work with Cas9 in targeting the three genes in both cultured fibroblasts and injected embryos.

### Prescreening for off-target effects of the sgRNAs in sheep fibroblasts

**Figure 2:**
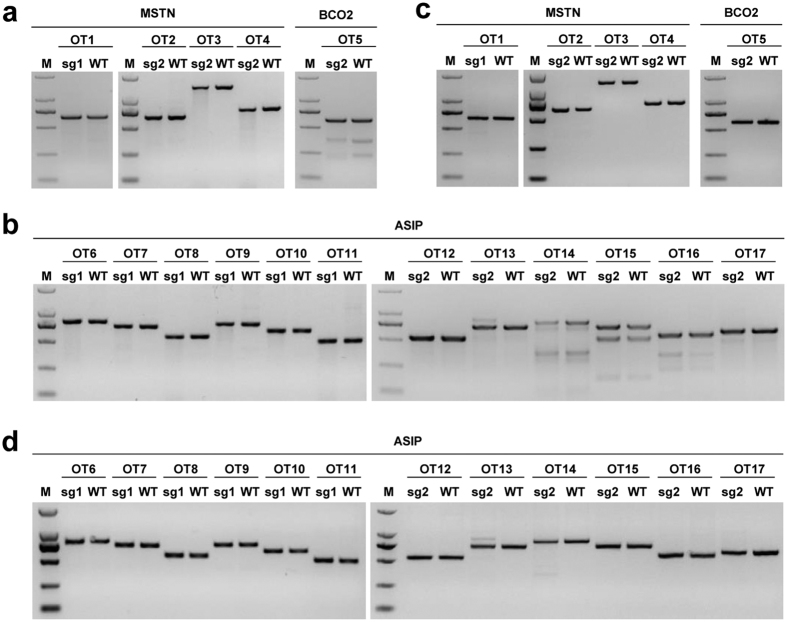
Detection of the sgRNA:Cas9-mediated off-target cleavage sites in sheep fibroblasts. The PCR products of the potential off-target sites in *MSTN*, *ASIP*, and *BCO2* sgRNA:Cas9 loci from sheep fibroblasts (**a**,**b**). 17 predicted off-target sites that were most homologous to *MSTN*, *ASIP*, and *BCO2* sgRNAs were named OT1 to OT17. (**c**,**d**) Detection of the sgRNA:Cas9- mediated off-target cleavage of *MSTN*, *ASIP*, and *BCO2* by using the T7EI cleavage assay. All PCR products from (**a**,**b**) were subjected to T7EI assay.

Off-target effects in the CRISPR/Cas9 system have remained a challenge since the establishment of this technology. To prevent the occurrence of off-target mutations in the Cas9 mediated sheep, before microinjection, we predicted putative the off-target effects of the selected sgRNAs and initially screened for the off-target mutations in fibroblasts obtained from Tan sheep. A total of 17 most potential off-target sites, including four for *MSTN*, 12 for *ASIP*, and one for *BCO2*, were predicted across the entire sheep genome (Supplementary Table S3). Among all the 17 off-target sites for the three target genes, no off-target mutations were detected in sheep fetal fibroblasts (Fig. 2a–d), indicating the high specificity of the sgRNAs used in the present study.

### Production of gene-modified sheep

After the successful validation of genetic modification in cultured sheep fibroblasts and injected embryos, we then generated gene-modified sheep with three genes that were simultaneous disrupted by co-injecting Cas9 mRNA (20 ng/μL) and sgRNAs (5 ng/μL for each sgRNA). A total of 613 sheep early embryos (one-cell stage) were surgically collected from 36 mated sheep using the superovulation approach as previously described^16^. A mixture of Cas9 mRNA and the sgRNAs was microinjected as described earlier. Collectively, 578 out of 588 injected embryos were transferred into 82 recipient females. After full-term (~150 days) pregnancy, five fetuses were aborted from four recipients, while 50 lambs, including seven stillbirths, were delivered from 30 recipients, and six lambs died immediately after birth, remaining 36 live lambs for further analysis (Table 2, Supplementary Table S4).

**Figure 3:**
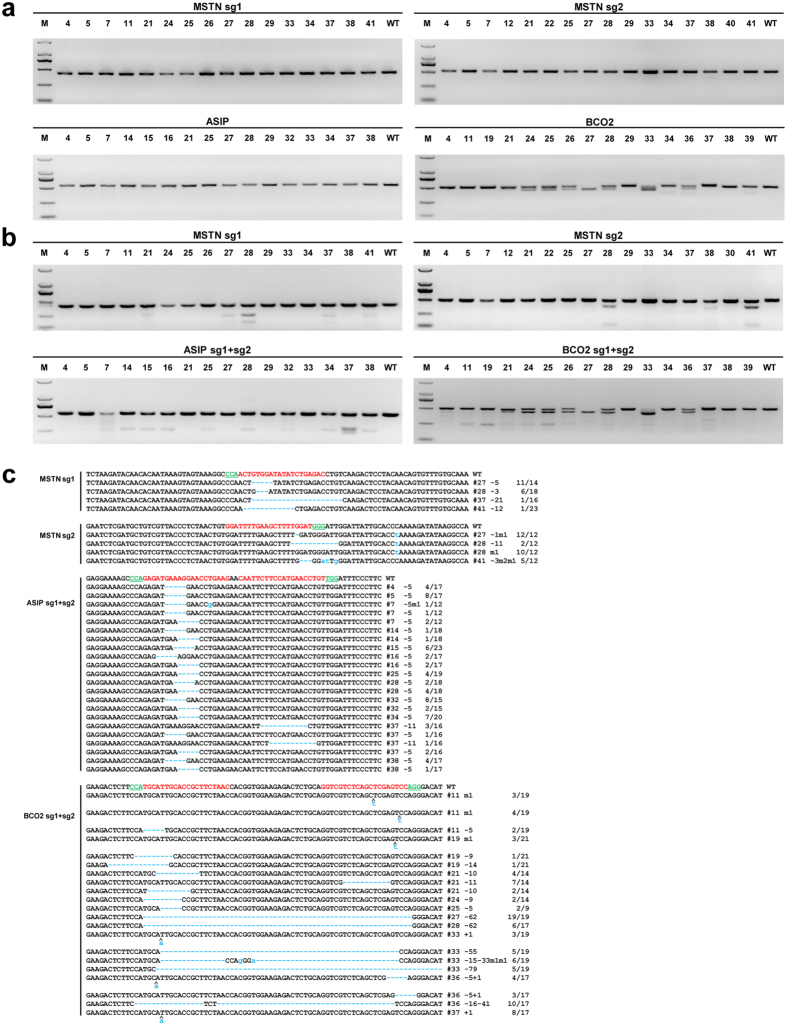
Evaluation of sgRNA:Cas9-mediated genetic modifications in lambs. (**a**) PCR products of the targeted region of the *MSTN*, *ASIP*, and *BCO2* genes of founder sheep co-microinjected with a mixture of Cas9 mRNA and sgRNAs. D2000 DNA Marker was used as a marker reference. (**b**) Detection of sgRNA:Cas9-mediated on-target cleavage of the *MSTN*, *ASIP* and *BCO2* genes by using a T7EI cleavage assay. All PCR products from (**a**) were subjected to the T7EI cleavage assay. (**c**) Sequencing results of the modified *MSTN*, *ASIP* and *BCO2* loci that were detected in the founder animals. Target sequences complementary to sgRNAs of targeted genes are in red text, while the PAM sequences are marked in green. The mutations are marked in blue, dashlines indicates deletions, and lowercase indicates insertions or replacements. Insertions (+), deletions (−), mutations (m) is shown to the right of each allele. The genotypes are shown to the right with the rates of total clones for TA-sequencing.

Genomic DNA of the 36 live lambs was extracted, and genotyping was performed through PCR amplification, T7EI assay, and TA-cloning sequencing. The additional bands, as observed by PCR amplification of the target region, were observed in some of the lambs (Fig. 3a), which suggested the occurrence of genetic modification. The PCR products from the samples of 54 individuals (5 aborted, 7 stillbirths, 6 dead animals, and 36 live animals) were subsequently subjected to the T7EI cleavage assay (Fig. 3b). Impressively, among 36 live animals, the cleavage products of the *MSTN* gene were detected in 10 lambs (27.8%), the *ASIP* gene in 12 infants (33.3%), and that of the *BCO2* gene in 10 lambs (27.8%), with only two founder animals (#28 and #37) demonstrated simultaneous inhibition of the three genes (Table 1, Supplementary Table S4). Sequencing of the PCR products indicated various types of indels (Fig. 3c), thereby confirming the genomic modifications.

All of the targeting results described above were derived from the blood samples. To evaluate whether the integration of the Cas9-mediated gene targeting was derived from the three germ layers, we collected various tissues including the heart, liver, spleen, lung, kidney, skin, muscle, testis and ovary from two aborted founders (#A14 and #A15). The T7EI cleavage bands of all the tissues from the same animal confirmed the occurrence of Cas9:sgRNA modification in the three genes of various sheep tissues (Supplementary Fig. S3a,b), thereby indicating that genetic modifications were extensive integrated into different tissues. We further characterized the mosaicism of the modified loci by sequencing the PCR products of randomly selected tissues, which identified variable mutations in ovary and skin at the *MSTN* sg1 locus (Supplementary Fig. S3c), thereby indicating that modification occurred at *MSTN*-sg1 locus during early embryogenesis.

### Phenotypic analysis of gene-modified sheep

**Figure 4:**
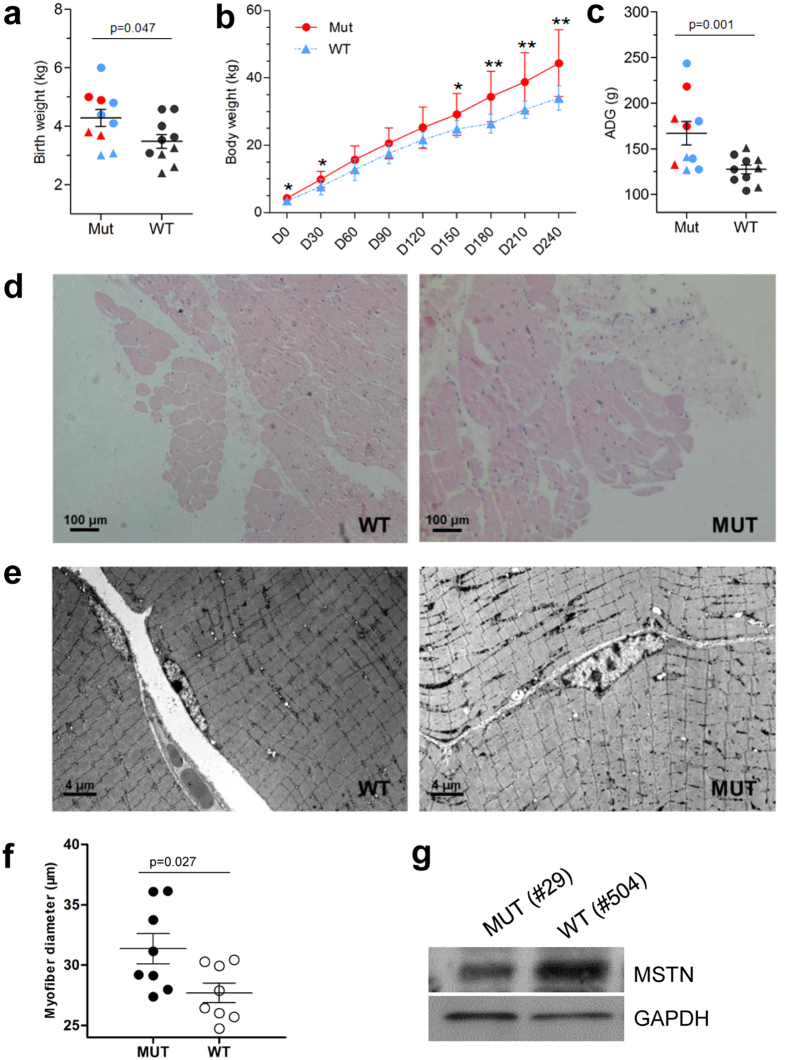
Phenotypic analyses of gene-modified sheep targeting *MSTN*. (**a**) Birth weight of the *MSTN*-disrupted and WT lambs. Red dots indicate *MSTN*-disrupted male founders; red triangles indicate *MSTN*-disrupted female founders. Blue dots indicate male founders with more than two genes (including *MSTN*) disrupted, blue triangles indicate female founders with more than two genes (including *MSTN*) disrupted. Black dots indicate male WT, blue triangles indicate female WT. (**b**) Body weight changes in *MSTN*-disrupted and WT sheep from D0 to D240. *p < 0.05, **p < 0.01, student’s *t*-test. (**c**) The average daily gain (ADG) of *MSTN*-disrupted and WT sheep from D0 to D240. (**d**) H&E staining shows the morphology of myofibers in the muscles of WT and *MSTN*-disrupted (MUT) (#29) sheep. (**e**) TEM analyses of muscle tissues of a mutant (#29) and WT sheep at D120. Scale bars: left = 2 μm. (**f**) The diameter of myofibers for a mutant (#29) and WT sheep. (**g**) Western blotting analysis using anti-MSTN and anti-GAPDH (loading control) antibodies in a *MSTN*-disrupted sheep (MUT(#29)) and WT (#504).

The 36 live-born founders are all alive at the time this paper was written. Laboratory analyses of key chemistry parameters using blood samples from both founders (n = 10) and wild-types (WT) animals (n = 10) showed that these were in good health conditions (Supplementary Table S5). Subsequently, we investigated whether Cas9-mediated precise genome editing led to phenotypic alterations in sheep that carried corresponding mutants, and examined the phenotypes of the gene-modified animals. Because the *MSTN* gene is a negative regulator of muscle growth in sheep, and *MSTN* mutant shows apparent muscle hypertrophy easy to check, we then carefully compared the body weight (BW) between the ten alive *MSTN*-disrupted animals and ten WT from D0 to D240. As expected, the average birth weight of *MSTN-*disrupted sheep was significantly higher than that of the WT (P < 0.05) (Fig. 4a, Supplementary Table S6). The BW of *MSTN*-disrupted lambs at D30 and D150 to D240 was remarkably higher than that of WT sheep (Fig. 4b, Supplementary Table S6). The BW of *MSTN*-disrupted male sheep at D240 was 1.29 fold higher than that of the controls (Supplementary Table S6). We further evaluated the average daily gain (ADG) in each animal from D0 to D240 and observed significant differences between *MSTN*-disrupted sheep and WT (P < 0.01) (Fig. 4c), indicating that the lambs with edited *MSTN* underwent accelerated postnatal growth.

Genome targeting of tissues representing three germ layers of the two aborted individuals (#A14 and #A15) indicated that mutations extensively occurred across various tissues, thereby providing samples for further analysis of mutation-mediated phenotypic changes. We collected muscles from two 150-day-old founders (#28 and #41). The T7EI assay showed cleavage bands of the *MSTN* gene in the muscles of both animals (Supplementary Fig. S4a,b). Sequencing further confirmed that the muscles were mutated at the cleaved site of variable mutation sizes in different clones from each animal (Supplementary Fig. S4c). Then, we further assessed the effects of genetic modification on the muscle development of *MSTN*-disrupted lambs by conducting hematoxylin-eosin (H&E) staining and transmission electron microscopy (TEM) analyses of muscle tissues from the corresponding Cas9-mediated sheep. Remarkably, the diameter of the myofibers of *MSTN*-disrupted lamb (#29, #28, and #41) was apparently larger than that of the WT (Fig. 4d,f, Supplementary Fig. S5). TEM analyses further confirmed that the myofiber diameter of *MSTN*-disrupted animals was larger than that in the control animals (Fig. 4e). We subsequently conducted Western blotting to confirm the decreased MSTN expression in *MSTN*-disrupted animals (Fig. 4g). These findings indicated that the larger size of muscle fibers in *MSTN*-deficient founders was attributable to the increase in BW.

### Germline transmission of mutant alleles

Germline transmission of mutations in target genes to the next generation is essential for expanding the population of gene-disrupted individuals. Because it takes at least 10–12 months for Tan sheep to reach sexual maturity, which is then followed by a ~150-day gestational period, we currently cannot perform breeding to determine germline transmission. However, the observed high frequency of targeted mutations in different tissues strongly suggests that Cas9-mediated genome targeting efficiently integrates into the sheep gonads. The abortive embryos allowed us to elucidate the mechanism of integration of Cas9-mediated genome targeting into the derivatives of different layers of embryo, thereby providing direct evidences to support our hypothesis that every tissue carries mutations (Supplementary Fig. S3).

**Figure 5:**
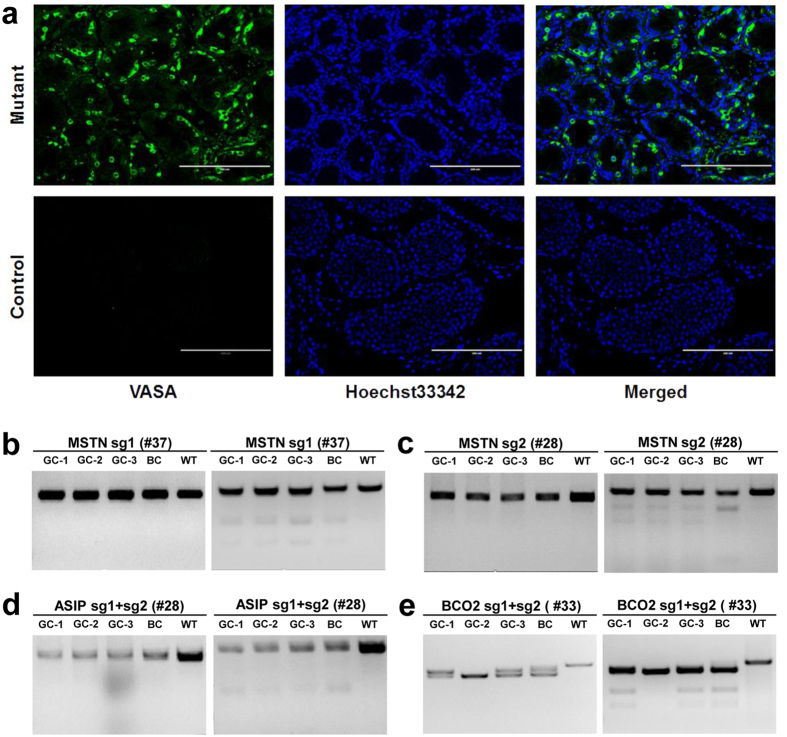
Detection of germline transmission in founder animals. (**a**) Immunostaining analysis of biopsied testis of the founder (#28) at 120-day-old, confirmed by germ cell specific marker VASA. Germ cells from the testis were stained with an anti-VASA antibody (green) and Hoechst 33342 (blue). VASA positive cells are germ cells. VASA negative cells were used as negative control. Scale bar = 200 μm. (**b**–**e**) PCR products of the targeted loci and detection of sgRNA:Cas9-mediated on-target cleavage in germ cells (GCs) and blood cells (BCs) from founders (#28, #33, and #37).

Furthermore, the existence of germ cells in the testis of founder #28 was supported by immunostaining with VASA, a germ cell specific marker (Fig. 5a). In addition, the germ cells from the testis of three founder animals (#28, #33, and #37) were biopsied and single germ cells from each animal were isolated for T7EI cleavage and PCR sequencing. By using T7EI cleavage assay and Sanger sequencing, we extensively analyzed targeted mutagenesis in both germ cells and blood cells. T7EI cleavage bands of the *MSTN* gene were observed in germ cells from sheep testis as well as blood cells (Fig. 5b–e). The sequencing results confirmed that the single germ cell from the testis harbored the same mutations as that of blood cells at the *MSTN* sg1 locus and *ASIP* sg1 and sg2 loci (Supplementary Fig. S6), indicating a high probability that Cas9-mediated target mutations in the founder animals will be transmitted to the next generations.

## Discussion

Recent studies have demonstrated that the type II CRISPR-Cas9 system of *Streptococcus pyogenes* holds great promise in functional genomic studies^13^, gene therapy^30^, and may thus be potentially applied to a wide variety of crops and animals with agriculturally valuable traits^12^,^31^. The CRISPR/Cas9 system demonstrates that site-specific gene modification can be efficiently achieved by co-injecting of Cas9 mRNA and sgRNAs that target multiple independent genes in sheep, which is a major livestock, as well as other model species utilized in biomedical studies. Two recent studies reported the generation of a single gene (*MSTN*)-disrupted sheep using the CRISPR/Cas9 system by zygote injection^18^,^19^. We tested the targeting efficacy of sgRNAs in sheep fibroblasts and zygotes injected with pooled sgRNAs, and the efficacy was as high as 50–80% in fibroblasts and 35–50% in injected zygotes. This success inspired us to assess its efficacy in lambs generated by microinjection, and the observed targeting efficacy of a single gene was ranged from 27.8–33.3%. Although we show that targeting multiple genes/loci is feasible in sheep, only two alive founders (2/36, 5.6%) with three genes disrupted were obtained in the present study, we are expecting further studies to achieve moderate improvements in gene targeting efficiency.

The phenotypic traits in animals are generally determined by multiple genes. The CRISPR/Cas9 system is characterized by multiplexity in genome targeting and has been successfully applied to multiple gene targets in different animals^13^,^32^,^33^, thereby suggesting its feasibility in simultaneously disrupting several genes in sheep. By injection of the mixture of six sgRNA targeting at *ASIP*, *BCO2*, and *MSTN*, we successfully mutated the three genes simultaneously at an efficiency of 5.6%, demonstrating multiplexity of CRISPR/Cas9-mediated genome editing, as well as the possibility for improving different traits determined by various genes in sheep.

The ultimate goal of gene editing in sheep is to change their phenotype. With our success in gene targeting, we carefully analyzed the resulting phenotype after *MSTN* targeting. We observed apparent BW increase as reported by other groups^6^,^19^, more interesting, BW measurements showed that the *MSTN*-disrupted sheep were heavier than controls. The ADG of *MSTN*-disruption lambs was 1.31-fold higher than that of control animals (167 g vs. 127 g) from D0 to D240 (Supplementary Table S6), which is consistent with the ADG results of *MSTN*-disrupted goats generated in our prevous study^16^. We further observed that the increases in BW and ADG were caused by muscle hypertrophy, which is characterized by enlarged myofibers, and is a typical trait of natural *MSTN* mutation. These results have allowed us us to improve the economically significant traits of sheep by CRISPR/Cas9-mediated gene editing.

Biosafety is one of the major concerns that are realted to transgenic farm animals because genome editing may result in harmful consequences^34^. To prevent possible off-target effects resulting from Cas9/sgRNA-mediated gene editing, we performed prescreening and demonstrated the specificity of the selected sgRNAs before microinjection. As expected, no detectable off-targets were induced in the founders prior to receiving the sgRNAs. These results indicated the feasibility of screening for off-targets prior to using the CRISPR/Cas9 system. Furthermore, we monitored the health status of 10 randomly selected founders from 36 live born animals by analyzing their blood chemistry parameters (Supplementary Table S5). The results showed that all live mutant animals were healthy, thereby providing further evidence of the reliability of animals produced by using the CRSIPR/Cas9 method^35^.

Germline transmission of knockout alleles to the next generation is essential to the expansion of founder populations. In accordance with a previous study^36^, sequencing of the genomic DNA from ovary and germ cells derived from the testis of gene-modified animals suggests that germline transmission in sheep that underwent Cas9-modfication is highly efficient. Majority of mutant alleles in founder animals are capable of germline transmission, regardless of whether these are mosaic, thus suggesting that the somatic mutations induced in the single-cell embryos were maintained in the germ cells with high fidelity. Nevertheless, we are expecting the subsequent breeding studies with several generations to provide solid results on germline transmission in both genotypes and phenotypes.

Taken together, we report here the successful targeting of three genes, thereby economical trait change in sheep by using a CRISPR/Cas9 system with improved efficacy and high specificity. These results suggested that this technique may facilitate the rapid improvement of livestock by targeting multiple genes with major functional roles, although the biosafety issue of the CRISPR/Cas9 technology need to be further clarified prior to its application to animal improvement.

## Materials and Methods

### Animals and phenotyping of founders

The animals used in the present study were maintained at the Ningxia Tianyuan Sheep Farm, Hongsibu, Ningxia Autonomous Region, China. All animals were handled in accordance with the Guidelines for the Care and Use of Laboratory Animals that was established by College of Animal Science and Technology, Northwest A&F University. All protocols involving the use of animals were approved by Northwest A&F University (Approval ID: 2014ZX08008002).

Founder animals were kept and managed under the same conditions, and fed the same feed throughout the study period. The feed was adjusted with respect to growth stages after weaning at D60. The BW of all tested animals was recorded at D0 and every 30 days until D240. Jugular venous blood samples were collected from randomly selected edited sheep (n = 10) and randomly selected control animals (n = 10) at D240 for blood chemistry analyses.

### sgRNA design

To construct the recombinant vector for preparation of sgRNA by *in vitro* transcription, the two complementary DNA oligos (Supplementary Table S7) were annealed to be double-stranded and subcloned into pUC57-T7-gRNA vector as described elsewhere^29^. Using the constructed recombinant vector which was completely linearized by PCR amplification as templates, sgRNAs were produced via *in vitro* transcription using MEGAshortscript kit (Ambion) and purified using MEGAClear kit (Ambion) following the recommendations of the manufacturer. Using the Cas9 mRNA *in vitro* transcription vector (Addgene No. 44758) as template, Cas9 mRNAs were produced and purified as described by Shen *et al*.^29^.

### Cas9/sgRNA efficacy test in sheep fibroblasts

Sheep fibroblasts were obtained from the trunk region of a 40-day-old sheep fetus and transported to the laboratory. The fibroblasts were cultured for five passages in DMEM medium (Gibco) supplemented with 10% FBS (Gibco) and 1% penicillin-streptomycin (Gibco) until 80–90% confluency, which were then used for transfection. The transfection procedure was performed using Lipofectamine2000 Reagent (Invitrogen) according to the manufacturer’s instructions. Briefly, fibroblasts were separately transfected with (1) *MSTN*-sg1 (0.8 μg), *MSTN*-sg2 (0.8 μg), (2) *ASIP*-sg1 (0.8 μg), *ASIP*-sg2 (0.8 μg), and *ASIP* (0.4 μg) + *ASIP* (0.4 μg), (3) *BCO2*-sg1 (0.8 μg), *BCO2*-sg2 (0.8 μg), and *BCO2* (0.4 μg) + *BCO2* (0.4 μg), along with 0.8 μg of Cas9 plasmid by Lipofectamine 2000 in a 24-well culture plate. 24 h after transfection, 10 mg/mL of blasticidine S hydrochloride was added to the medium (1:1,000 dilution) and incubated for 24 h. Genomic DNA was extracted from fibroblasts at 72 h after transfection using a saturated solution of phenol and chloroform, then the DNA was precipitated with alcohol and sodium acetate. Subsequently, a T7EI cleavage assay was performed as described by Shen *et al*.^29^. Briefly, the targeted fragments were amplified using PrimerSTAR HS DNA polymerase (TaKaRa, DR010A), then purified with a PCR cleanup kit (Axygen, AP-PCR-50). The primers for amplifying the targeted fragments of the *MSTN*, *ASIP*, and *BCO2* genes are listed in Supplementary Table S8. Purification of PCR products, T7EI cleavage assay, and Sanger sequencing were performed according to Wang *et al*.^16^.

### Detection of off-target sites in sheep fibroblasts

We predicted the potential off-target sites using SeqMap^37^, in order to determine the site-specific cleavage of the CRISPR-Cas9 system in sheep fibroblasts. The procedures for searching off-target sites were performed as previously described^16^. The selected potential off-target loci were first amplified using genomic DNA from cultured sheep fibroblasts. The PCR products were then subjected to a T7EI cleavage assay. The off-target loci that yield typical cleavage bands were chosen as candidates for off-target detection in animals generated by CRISPR/Cas9.

### Zygote collection and production of gene-modified sheep

Healthy ewes (3–5 years old) with regular estrus cycles served as donors for zygote collection. Zygote collection and treatment of donors were conducted as earlier described in goats^16^. One-cell stage zygotes (around 10–16 h after the last insemination) were surgically collected and were immediately transferred into a TCM199 medium (Gibco). Cas9 mRNA (20 ng/μL) and sgRNAs (5 ng/μL for each sgRNA) targeting *MSTN*, *ASIP* and *BCO2* were mixed and injected into the cytoplasm of fertilized oocytes using an Eppendorf FemtoJet system. The injection pressure, injection time and compensatory pressure were 45 kpa, 0.1 s and 7 kpa, respectively. Microinjection was conducted in a manipulation medium TCM199 on the heated platform of an Olympus micromanipulation system ON3. After injection, the zygotes were cultured using Quinn’s Advantage Cleavage Medium and Blastocyst Medium (Sage Biopharma) for 168 h at 38.5 °C, and blastocyst embryos were preserved in PBS, followed by PCR amplification and T7EN1 analysis. The rest of the zygotes were cultured in Quinn’s Advantage Cleavage Medium (Sage Biopharma) for 24 h at 37 °C and then transferred to Quinn’s Advantage Blastocyst Medium (Sage Biopharma) at 37 °C, 5% concentration of CO_2_ and saturated humidity.

We determine the surrogates according to their natural estrus cycles. Around 3–4 divisive embryos were transferred into the ampullary-isthmic junction of the oviduct of the recipient ewes. Pregnancy normally takes about 150 days in Tan sheep, and was determined based on the observation of estrus behaviors in every ovulation cycle.

### T7EI cleavage assay and sequencing

Different samples (injected zygotes or tissues) were collected and digested in lysis buffer (0.4 M NaCl, 2 μM EDTA, 1% SDS, 10 μM Tris-HCl, and 100 μg/mL Proteinase K). The genomes of the embryos were amplified using a REPLI-g Single Cell kit (QIAGEN, 150343), whereas genomic DNA was extracted from tissues isolated from lysate by using phenol-chloroform, and recovered by alcohol precipitation. A T7EI cleavage assay was performed as described by Shen *et al*.^29^. Briefly, targeted fragments were amplified using PrimerSTAR HS DNA polymerase (TaKaRa, DR010A), then purified with a PCR cleanup kit (Axygen, AP-PCR-50). The primers used in the amplification of targeted fragments of the three genes are presented in Supplementary Table S8. The purified PCR products were denatured and re-annealed in NEBuffer 2 (NEB) using a BioRad thermocycler. The PCR products were digested with T7EI (NEB, M0302L) for 30 min at 37 °C and separated by 2.5% agarose gel electrophoresis. The PCR products with mutations detected by T7EI cleavage assay were sub-cloned into a T vector (Takara, D103A). For each sample, the colonies were randomly picked and sequenced by using an M13F (−47) primer (M13F (−47): 5′-CGC CAG GGT TTT CCC AGT CAC GAC-3′).

### H&E staining and TEM analyses

Muscle tissues collected from the hips of the *MSTN*-disrupted (#29) and WT sheep with 150-day-old were biopsied. Tissue biopsies were immediately fixed with 4% paraformaldehyde at 4 °C overnight, then embedded in paraffin using standard laboratory procedures. After cutting the samples into 2-μm slices, these were stained with H&E. Tissue sections were dewaxed, rehydrated, and stained using standard immunohistochemistry protocols. Eight representative images of muscles from founder #29 treated with H&E staining were selected, and the diameter of myofibers was measured using the AxioVision 4.2 software as previously described^38^.

The muscle samples were prepared for TEM analysis as previously described^39^. Briefly, the muscle sections were post-fixed with 1% (w/v) glutaraldehyde in 0.1 M PBS (pH 7.4) for 10 min, then washed in distilled water. After enhancement with an HQ Silver Kit (Nanoprobes), the sections were incubated at room temperature with 1:50-diluted Elite ABC Kit (Vector, Burlingame, CA) in 0.05 M TBS for 6 h, then further incubated at room temperature with 0.05 M Tris-HCl (pH 7.6) containing 0.02% (w/v) 3,30-diaminobenzidine tetrahydrochloride (DAB) and 0.003% (v/v) H_2_O_2_ for 20 min. Subsequently, the sections were placed in 0.1 M PBS (pH 7.4) containing 1% (w/v) O_s_O_4_ for 1 h and then counterstained with 1% (w/v) uranyl acetate in 70% ethanol for 1 h. Following dehydration, the sections were mounted on silicon-coated glass slides and flat embedded in epoxy resin (Durcupan, Buchs, Switzerland). Once the resin had polymerized, small pieces of tissues were cut from the flat-embedded sections, and selected tissue pieces were cut into 60-nm-thick sections using an ultramicrotome (Reichert-Nissei Ultracut S). Sections were examined with a JEM-1230 electron microscope (JEOL) and captured by a Gatan 832 CCD (Gantan).

### Western blotting

Total proteins were extracted from biopsied sheep muscles using a ProteoJET Membrane Protein Extraction Kit (Fermentas). Protein was then quantified using a Bradford assay. Equal amounts of soluble protein were separated by SDS/PAGE and transferred onto a polyvinylidene difluoride membrane (Roche). Immunoblotting was conducted using antibodies specific for MSTN (1:250, Sigma-Aldrich) and GAPDH (1:1,000, Sigma-Aldrich). Primary antibodies were visualized using a Fluorescence Imager system (Sagecreation). Variations in sample loading were estimated by densitometry and corrected based on the GAPDH band intensities.

### Germline transmission detection

The biopsied testis of founder #28 (120 days old) was examined by immunostaining with VASA, which is a germ cell-specific marker. Immunofluorescence staining was conducted as previously described. Briefly, rat anti-Daphnia VASA antibodies were raised against bacterially expressed N-terminal region of the VASA protein (residues 1–332) and affinity purified for immunostaining. A dilution of 1:500 of the primary antibody (1:500, Abcam) was used for most of the reactions. Germ cells from the testis of three founders (#28, #33, and #37) were obtained by using biopsy forceps (Olympus FB-11K). The genomes of the germ cells were amplified using a REPLI-g Single Cell kit (QIAGEN, 150343). The PCR products with mutations were detected by using the T7EI cleavage assay, followed by Sanger sequencing.

## Additional Information

**How to cite this article**: Wang, X. *et al*. Multiplex gene editing via CRISPR/Cas9 exhibits desirable muscle hypertrophy without detectable off-target effects in sheep. *Sci. Rep.* **6**, 32271; doi: 10.1038/srep32271 (2016).

## Supplementary Material

Supplementary Information

## Figures and Tables

**Table 1:** The efficacy of Cas9-mediated modifications in sheep fibroblasts, embryos and founder animals.

sgRNA	Fibroblasts	Injected embryos	Tested live lambs
*MSTN-sg1*	8/13 (61.5%)	7/20 (35.0%)	10/36 (27.8%)
*MSTN-sg2*	12/15 (80.0%)		
*ASIP-sg1* and *2*	6/12 (50.0%)	10/20 (50.0%)	12/36 (33.3%)
*BCO2- sg1* and *2*	5/7 (71.4%)	10/20 (50.0%)	10/36 (27.8%)
*MSTN*&*ASIP*&*BCO2*	—	4/20 (20%)	2/36 (5.6%)

**Table 2:** Summary of production of gene-modified sheep via the CRISPR-Cas9 system.

Number of donors for zygote collection	Number of collected embryos	Cas9-sgRNA injected embryo (one-cell stage)		Recipient	Aborted	Stillbirth	Newborn	
		Injected	Transferred (two-cell embryos)				Dead	Live
36	613	588	578	82	5	7	6	36
