# Supplementary material for: Multiplex gene editing via CRISPR/Cas9 exhibits desirable muscle hypertrophy without detectable off-target effects in sheep
Source: Sci Rep. 2016 Aug 26;6:32271. doi: 10.1038/srep32271 (PMC4999810; doi:10.1038/srep32271)
Supplement: Supplementary Information [file srep32271-s1.doc]

**Heritable multiplex gene editing via CRISPR/Cas9 exhibits desirable muscle hypertrophy without detectable off-target effects in sheep**

Xiaolong Wang^1§^, Yiyuan Niu^1§^, Jiankui Zhou^2,3§^, Honghao Yu^4§^, Qifang Kou^5^, Anmin Lei^6^, Xiaoe Zhao^6^, Hailong Yan^1,4^, Bei Cai^1^, Qiaoyan Shen^6^, Shiwei Zhou^1^, Haijing Zhu^4^, Guangxian Zhou^1^, Wenzhi Niu^5^, Jinlian Hua^6^, Yu Jiang^1^, Xingxu Huang^2,3*^, Baohua Ma^6*^, Yulin Chen^1*^

^1^College of Animal Science and Technology, ^6^College of Veterinary Medicine, Northwest A&F University, Yangling 712100, China.

^2^MOE Key Laboratory of Model Animal for Disease Study, Model Animal Research Center of Nanjing University, National Resource Center for Mutant Mice, Nanjing 210061, China.

^3^School of Life Science and Technology, ShanghaiTech University, Shanghai 201210, China.

^4^College of Life Science, Yulin University, Yulin 719000, China.

^5^Ningxia Tianyuan Sheep Farm, Hongsibu, 751999, China.

^§^These authors contributed equally to this work.

*Correspondence: [chenyulin@nwafu.edu.cn](mailto:chenyulin@nwafu.edu.cn) (Y.C.), [mabh@nwafu.edu.cn](mailto:mabh@nwafu.edu.cn) (B.M.), huangxx@shanghaitech.edu.cn (X.H.). (X.H.).

**Running title:** CRISPR/Cas9 and sheep gene targeting

**
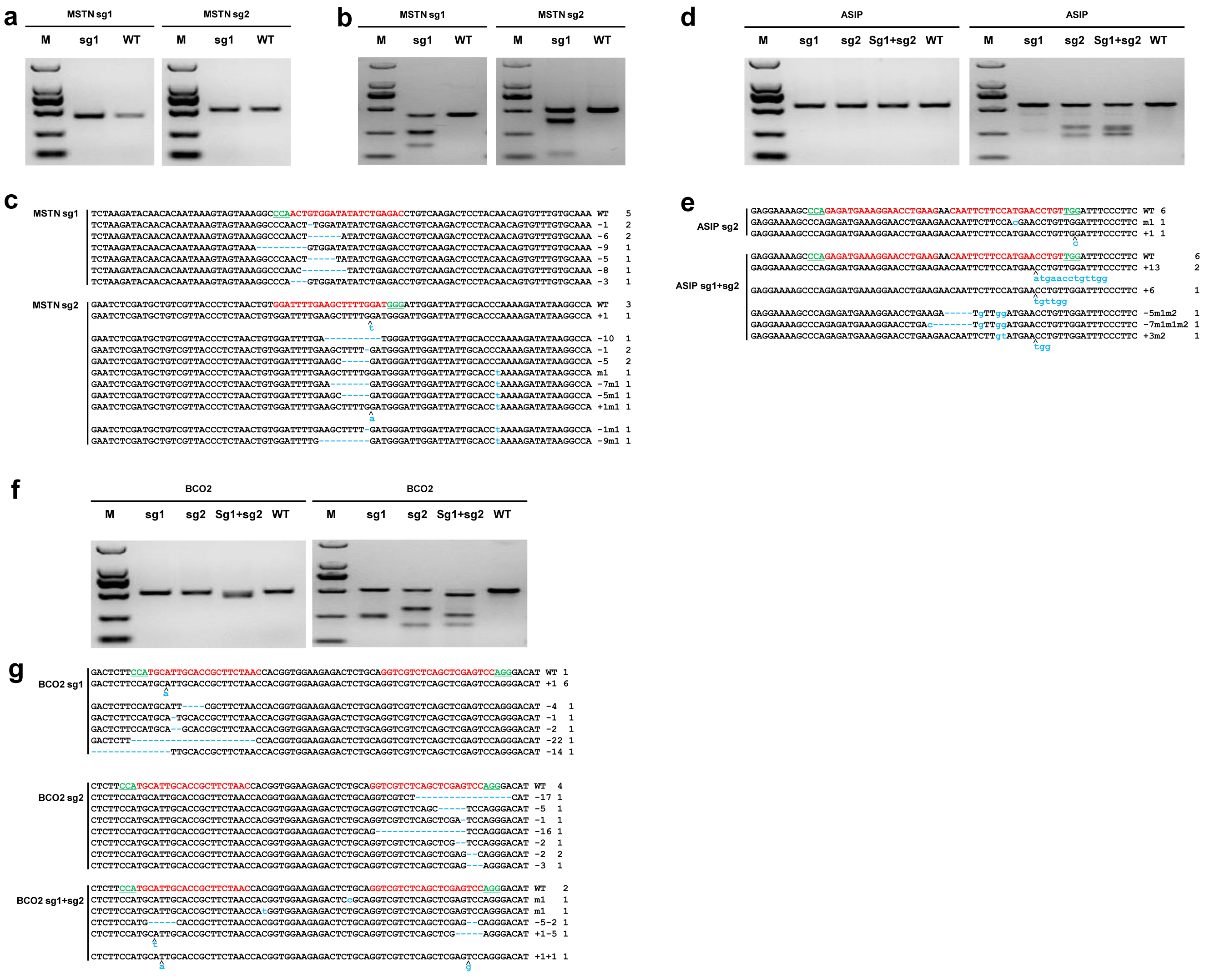
**

**Supplementary Fig. S1 Evaluation of sgRNA:Cas9-mediated genetic modifications in sheep fibroblasts.**

(a) PCR products of the targeted region of *MSTN* from sheep fibroblasts transfected with Cas9 and MSTN sgRNAs. (b) Detection of sgRNA:Cas9-mediated on-target cleavage of MSTN by T7E1 cleavage assay. PCR products from (a) were subjected to T7E1 cleavage assay. (c) Sequences of modified MSTN alleles. (d) PCR products and T7E1 cleavage of the targeted region of ASIP (d) and BCO2 (f) from sheep fibroblasts. (c) Sequencing results of modified ASIP (e) and BCO2 (g) alleles. Target sequences complementary to sgRNAs of target genes are in red text; the mutations are blue, lower case; insertions (+), deletions (-) or mutation (m) are shown to the right of each allele.


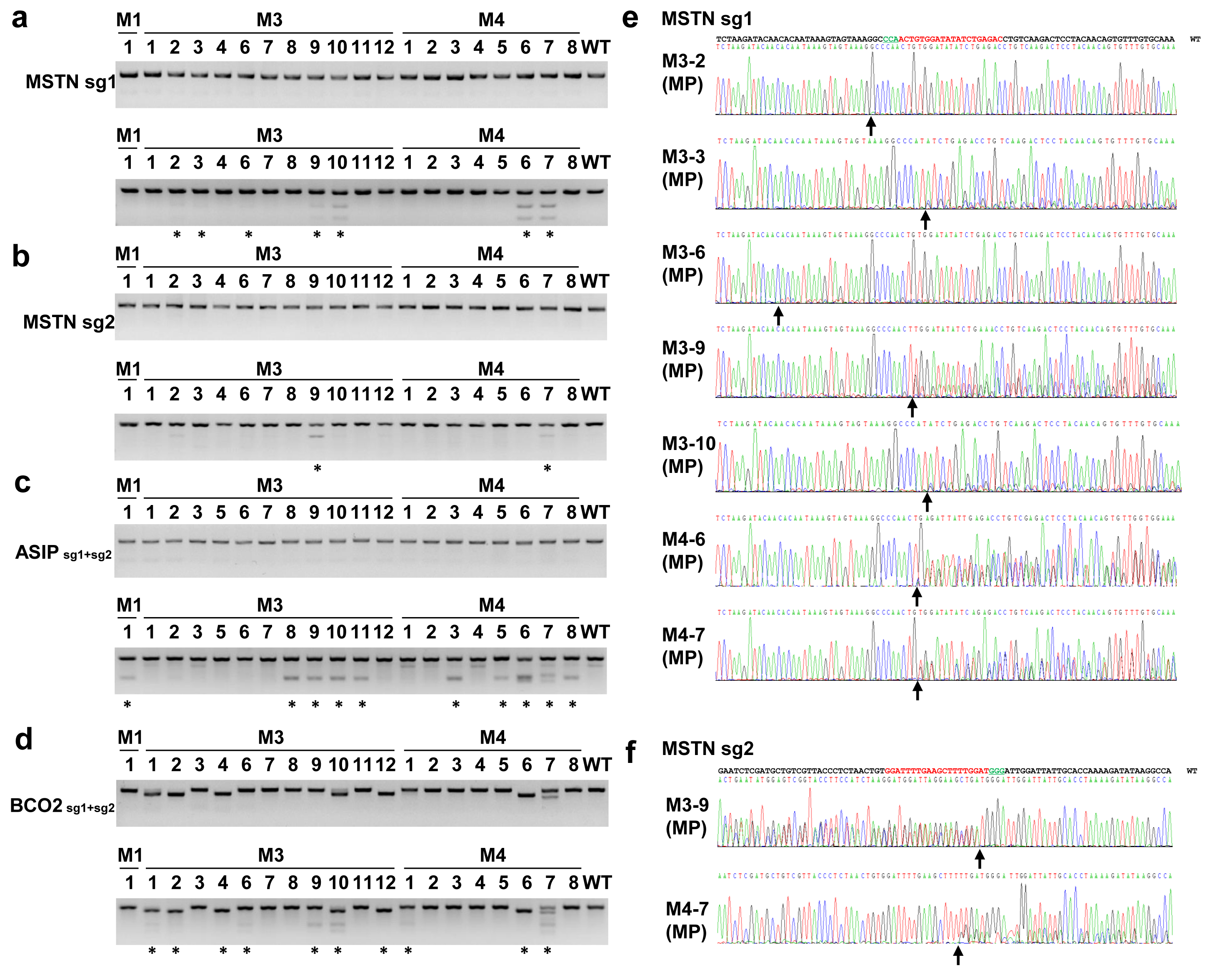


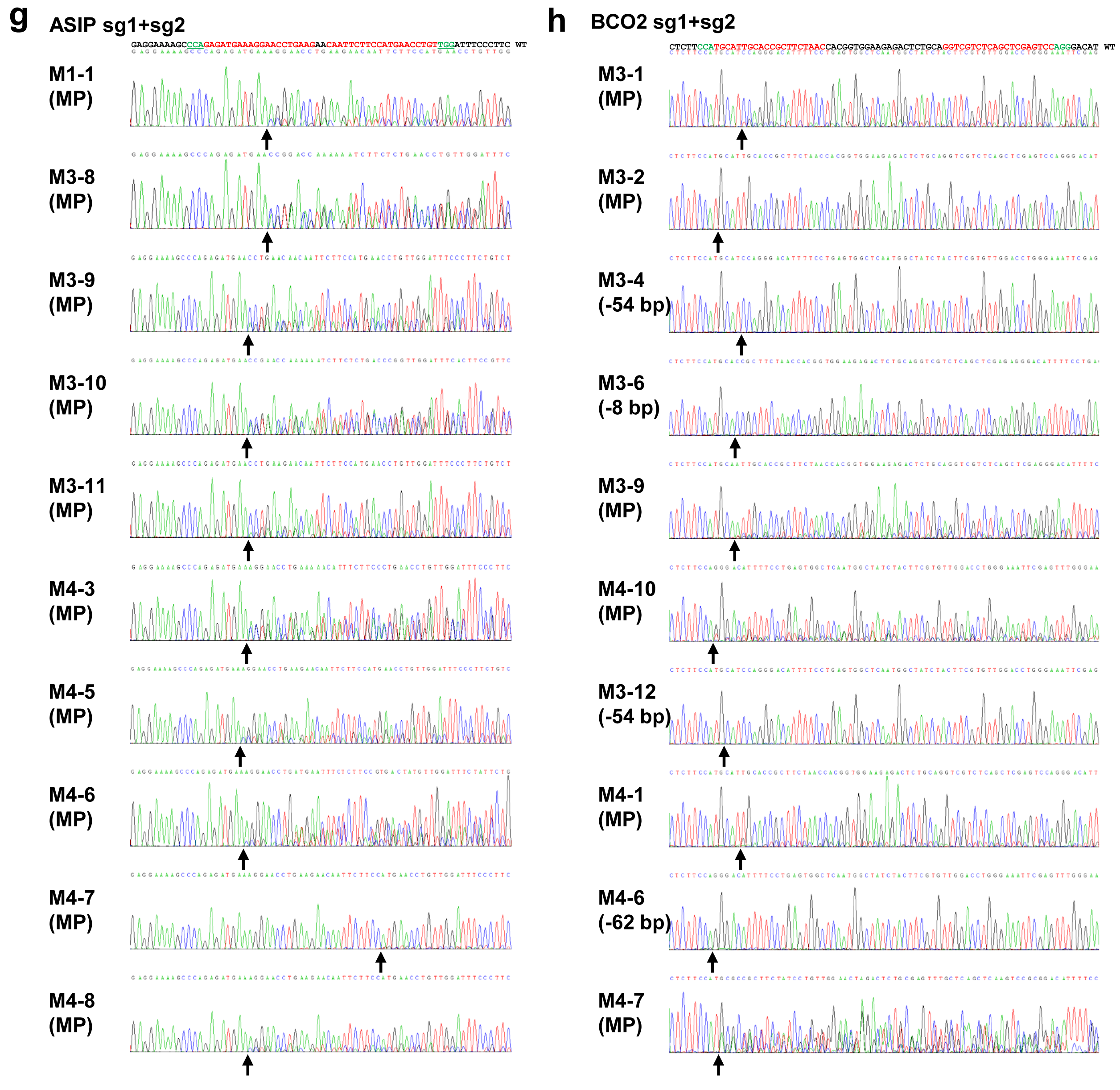
**Supplementary Fig. S2 Evaluation of sgRNA:Cas9-mediated genetic modifications in the injected embryos of sheep.** **(a-d)** PCR products and T7E1 cleavage assay of the targeted region of *MSTN, ASIP,* and *BCO2* from injected embryos. M1, M3, and M4 indicate different donors for embryo collection. Asterisks represent positive embryos. **(e-h)** Sanger sequencing of modified loci was detected in positive embryos. Arrows indicate the sites of modified loci, MP, multiple peaks.

**
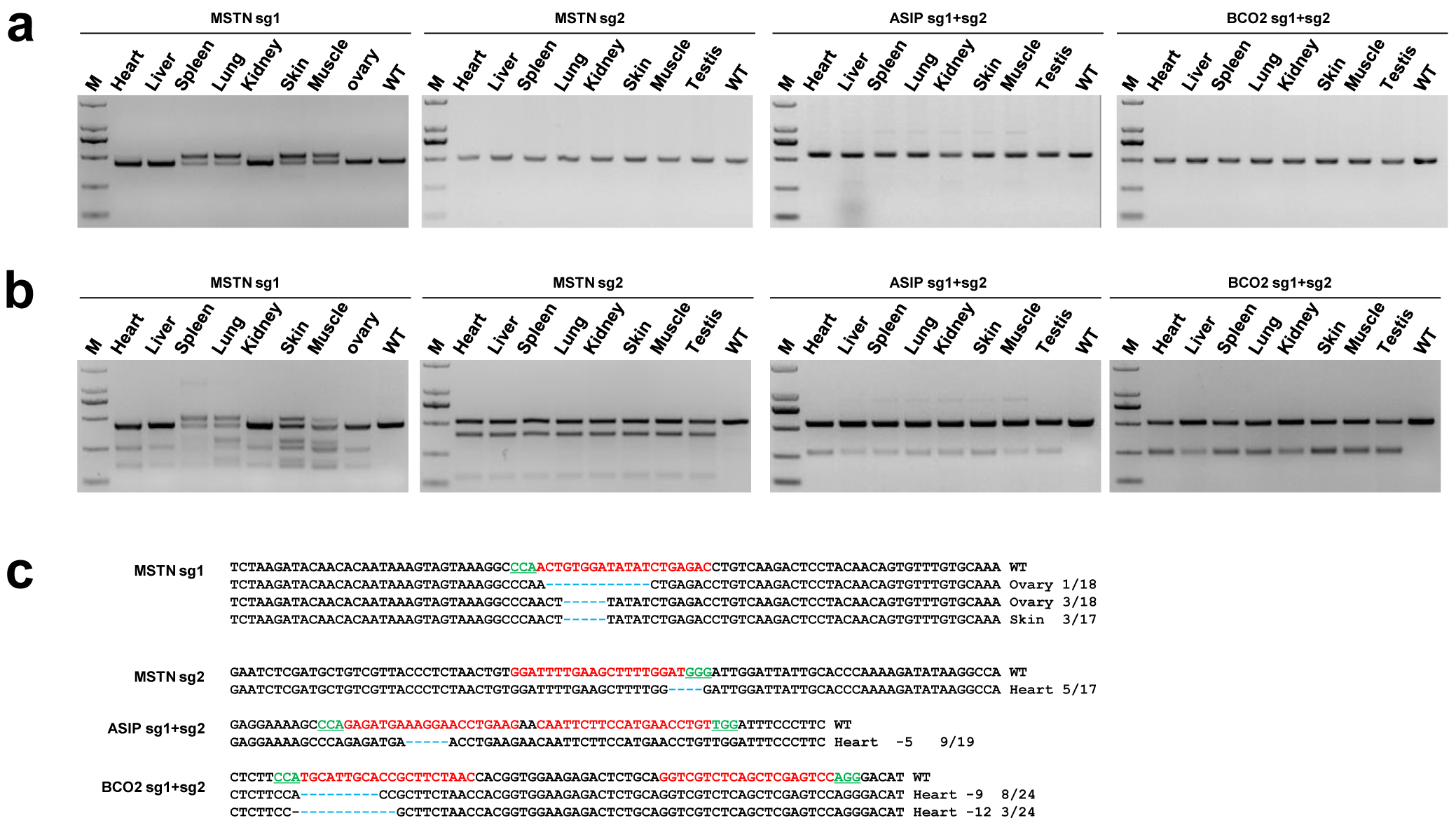
**

**Supplementary Fig. S3. Detection of sgRNA:Cas9-mediated targeting in different tissues.**

(a) Aborted individual #A15 was chose for tissue distribution analysis of on-target mutations of *ASIP*, *BCO2* and *MSTN*-sgRNA2. Aborted individual #A14 was chose for tissue distribution analysis of on – target mutations of *MSTN*-sgRNA1. (b) Detection of sgRNA:Cas9-mediated on-target cleavage *ASIP, BCO2* and *MSTN* by T7E1 cleavage assay. All PCR products from (a) were subjected to T7E1 cleavage assay. All the samples were digested by T7E1, suggesting that all the seven tested tissues carrying *ASIP, BCO2* and *MSTN* mutations. (c) Sanger sequencing of modified loci was detected in the randomly selected tissues (ovary and skin for *MSTN* sg1, heart for *MSTN* sg2, *ASIP* and *BCO2*) of founders.

**
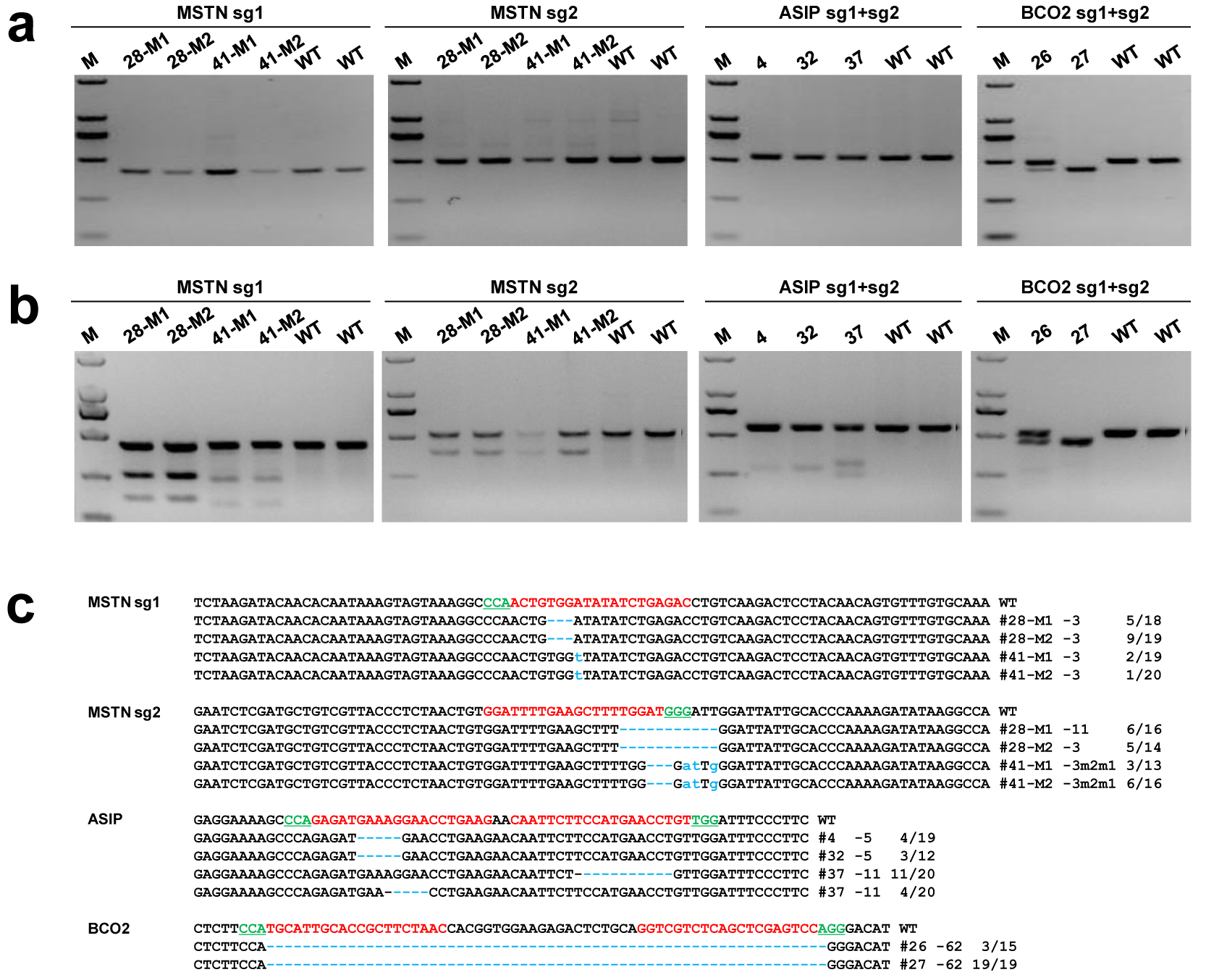
**

**Supplementary Fig. S4. Genotypes of three target genes in distinct tissues of founder animals.**

(a) PCR products of the targeted region of *MSTN, ASIP,* and *BCO2* from founder animals #28 and #41. Muscles of founder animals #28 and #41 were used to amplify the *MSTN* gene*,* skin tissues of founder animals #4, #32, and #37 were used to amplify the *ASIP* gene, and adipose tissues from founder animals #26 and #27 were used to amplify the *BCO2* gene. M1 and M2 indicate muscle tissues sampled from different areas of the same animals. (b) Detection of sgRNA:Cas9-mediated on-target cleavage of *MSTN, ASIP,* and *BCO2* by T7E1 cleavage assay. All PCR products from (a) were subjected to T7E1 cleavage assay. (c) Sequencing results of modified *MSTN, ASIP,* and *BCO2* loci detected in the tissues of founders.


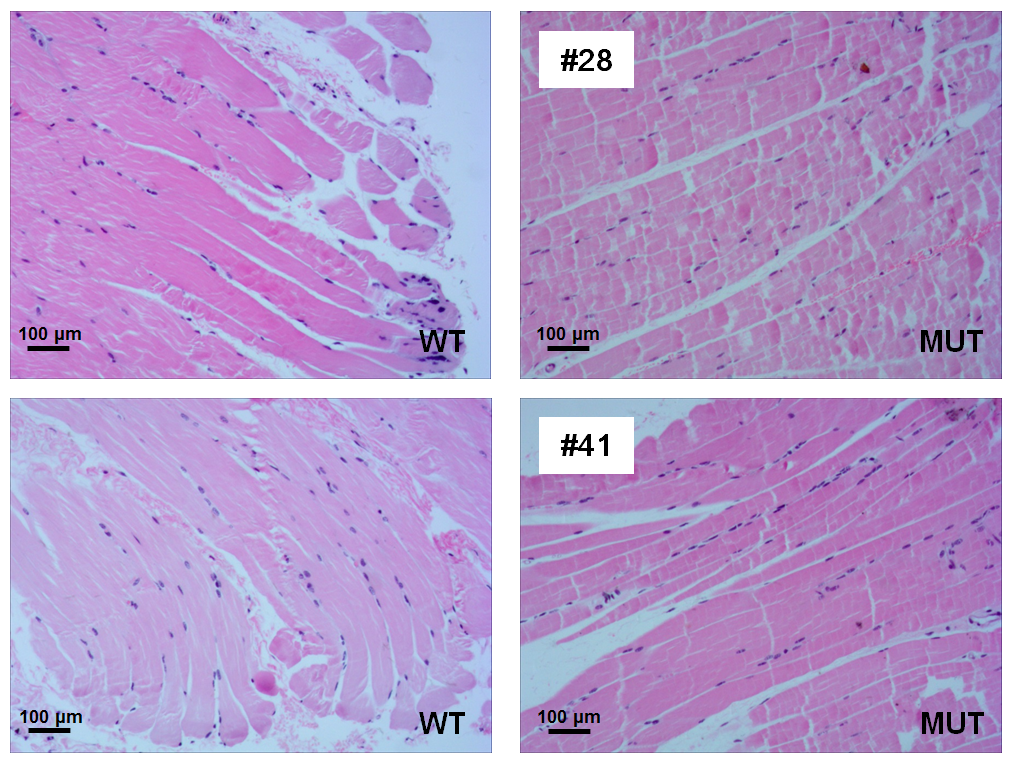


**Supplementary Fig. S5. (d)** H&E staining shows the morphology of myofibers in the muscles of WT and *MSTN*-disrupted (MUT) (#28 and #41) sheep.

**
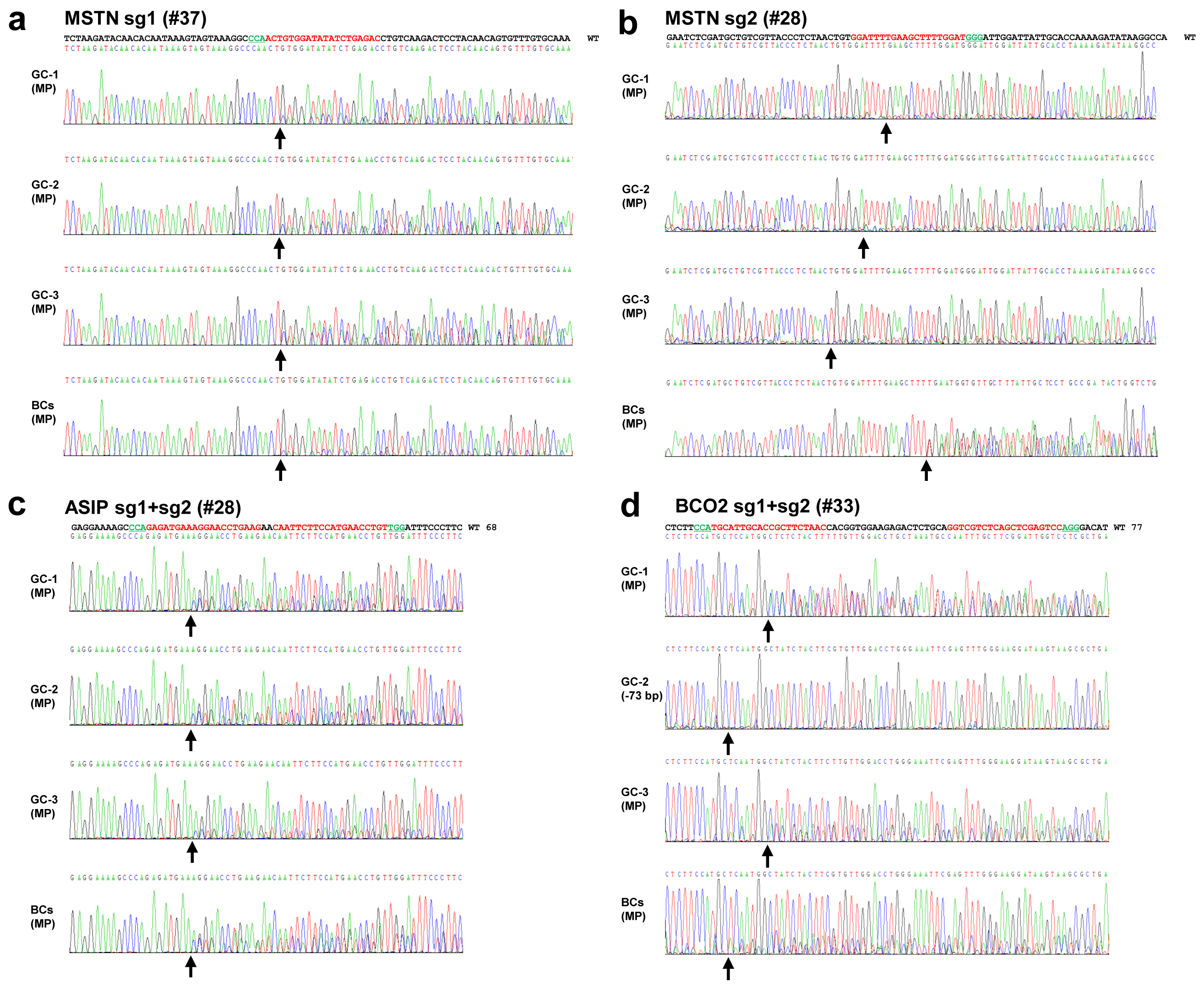
**

**Supplementary Fig. S6.** Sanger sequencing confirmed that the mutations in germ cells (GC) and blood cells (BC) of modified loci in *MSTN*, *ASIP*, and *BCO2*. Arrows indicate the sites of modified loci, MP, multiple peaks.

**Supplementary Table S1** sgRNA sequences and target sites.

| **sgRNA** | **Targeting site** | **Location** | **Strand** |
| --- | --- | --- | --- |
| MSTN sgRNA-1 | GTCTCAGATATATCCACAGTTGG | Chr2:118146737-118146759 | - |
| MSTN sgRNA-2 | GGATTTTGAAGCTTTTGGATGGG | Chr2:118149167-118149192 | + |
| ASIP sgRNA-1 | CTTCAGGTTCCTTTCATCTCTGG | Chr13:63047412-63047434  Chr13:63237519-63237541 | -  - |
| ASIP sgRNA-2 | CAATTCTTCCATGAACCTGTTGG | Chr13:63047437-63047459  Chr13:63237544-63237566 | +  + |
| BCO2 sgRNA-1 | GTTAGAAGCGGTGCAATGCATGG | Chr15:21947439-21947461 | - |
| BCO2 sgRNA-2 | GGTCGTCTCAGCTCGAGTCCAGG | Chr15:21947483-21947505 | + |

Coordinates of sgRNA target sites are based on the sheep genome assembly Oar_v3.1.

**Supplementary Table S2** Sequencing and T7E1 analysis summary of on-target on fibroblasts.

| No. | T7E1 | Sequenced Colonies | Mutations^a^ |
| --- | --- | --- | --- |
| MSTN-sgRNA-1 | positive | 2 | -1 |
|  |  | 2 | -6 |
|  |  | 1 | -9 |
|  |  | 1 | -5 |
|  |  | 1 | -8 |
|  |  | 1 | -3 |
| MSTN-sgRNA-2 | positive | 1 | +1 |
|  |  | 1 | -10 |
|  |  | 2 | -1 |
|  |  | 2 | -5 |
|  |  | 1 | m1 |
|  |  | 1 | -7m1 |
|  |  | 1 | -5m1 |
|  |  | 1 | +1m1 |
|  |  | 1 | -1m1 |
|  |  | 1 | -9m1 |
| ASIP-sgRNA-1 | negative | - | - |
| ASIP-sgRNA-2 | positive | 1 | m1 |
|  |  | 1 | +1 |
| ASIP-sgRNA-1 and 2 | positive | 2 | +13 |
|  |  | 1 | +6 |
|  |  | 1 | -5m1m2 |
|  |  | 1 | -7m1m1m2 |
|  |  | 1 | +3m2 |
| BCO2-sgRNA-1 | positive | 6 | +1 |
|  |  | 1 | -4 |
|  |  | 1 | -1 |
|  |  | 1 | -2 |
|  |  | 1 | -22 |
|  |  | 1 | -14 |
| BCO2-sgRNA-2 | positive | 1 | -17 |
|  |  | 1 | -5 |
|  |  | 1 | -1 |
|  |  | 1 | -16 |
|  |  | 1 | -2 |
|  |  | 2 | -2 |
|  |  | 1 | -3 |
| BCO2-sgRNA-1 and 2 | positive | 1 | m1 |
|  |  | 1 | m1 |
|  |  | 1 | -5-2 |
|  |  | 1 | +1-5 |
|  |  | 1 | +1+1 |

^a^Insertions (+), deletions (−), mutations (m).

**Supplementary Table S3** List of predicted off-target sites.


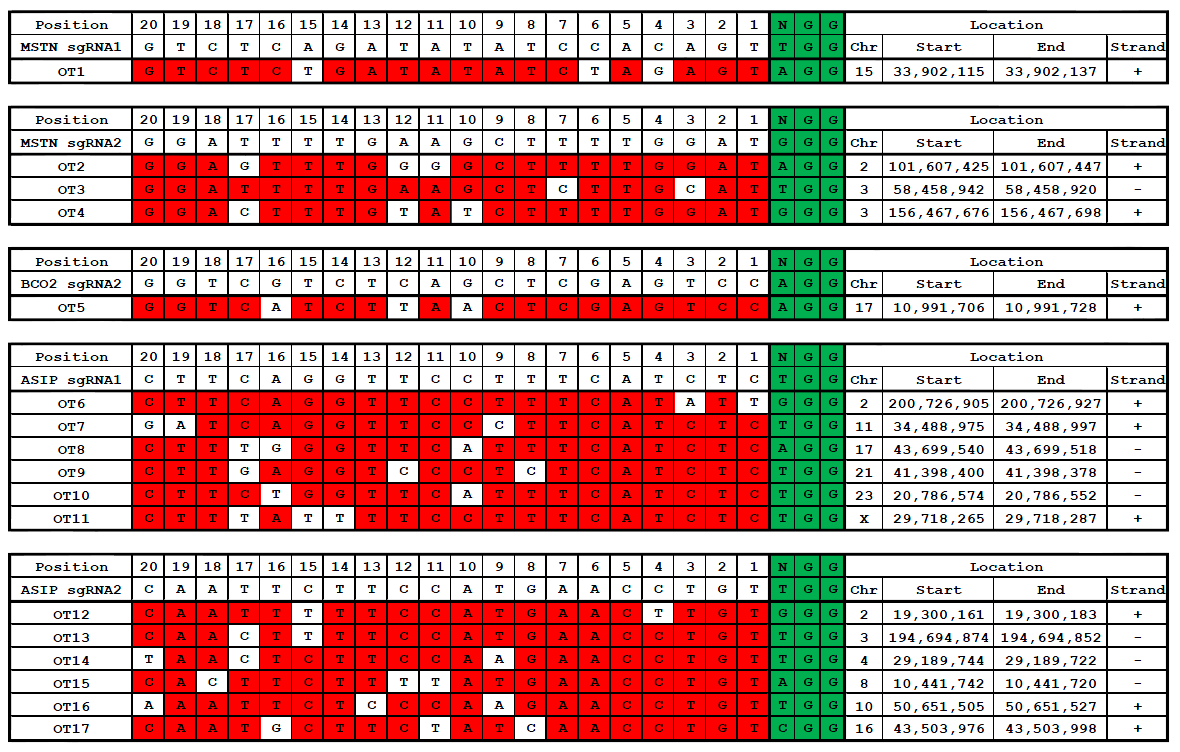


Nucleotides identical to sgRNA are shaded in red. PAM is shaded in green.

**Supplementary Table S4** Summary of delivery status and genetic modification of animals generated by CRISPR/Cas9.

| **Surrogate ID** | **Lamb ID at farm** | **Lab ID (#)** | **Gender** | **Delivery status** | | | | **Gene targeting** | | | | **Comments** |
| --- | --- | --- | --- | --- | --- | --- | --- | --- | --- | --- | --- | --- |
|  |  |  |  | **Gestation**  **length (d)** | **Delivery Weight (kg)** | **Delivery type** | **Offspring Status** | ***MSTN*_sg1** | ***MSTN*_sg2** | ***ASIP*** | ***BCO2*** |  |
| 1100968 | 1100968_1 | A4 | M | 127 | 2.0 | Aborted | Dead |  |  |  |  |  |
|  | 1100968_2 | A5 | F |  | 1.5 | Aborted | Dead |  |  |  |  |  |
| 090546 | 090546_1 | A3 | M | 125 | 1.2 | Aborted | Dead |  |  |  |  |  |
| 1100526 | 1100526_1 | A7 | F | 138 | 2.5 | Aborted | Dead |  |  |  |  |  |
| 1100714 | 1100714_1 | A12 | M | 141 | 2.1 | Aborted | Dead |  |  |  |  |  |
| 090556 | 1500505 | 4 | M | 153 | 3.4 | Natural | Live |  |  |  |  |  |
|  | 1500476 | A1 | F |  | 3.2 | Natural | Dead |  |  |  |  | Dead 1.5 days after birth |
| 1302058 | 1500415 | 5 | M | 150 | 3.8 | Natural | Live |  |  |  |  |  |
|  | 1500411 | A2 | M |  | 4.0 | Natural | Dead |  |  |  |  | Dead 2 days after birth |
| 110580 | 1500468 | 7 | F | 151 | 5.5 | Natural | Live |  |  |  |  |  |
| 100648 | 100648_1 | A8 | F | 153 | 2.0 | Natural | Dead |  |  |  |  | Weak body conditions, dead immediately after birth |
| 100556 | 100556_1 | A13 | M | 154 | 4.9 | Stillbirth | Dead |  |  |  |  | Stillbirth by large body |
| 1300244 | 1500444 | 11 | F | 153 | 4.0 | Natural | Live |  |  |  |  |  |
|  | 1500484 | 12 | F |  | 3.8 | Natural | Live |  |  |  |  |  |
| 0100094 | 1500442 | 13 | F | 151 | 4.6 | Natural | Live |  |  |  |  |  |
| 03501 | 1500488 | 14 | F | 155 | 3.3 | Natural | Live |  |  |  |  |  |
|  | 1500490 | 15 | F |  | 3.0 | Natural | Live |  |  |  |  |  |
| 1300452 | 1500522 | 16 | F | 155 | 4.45 | Natural | Live |  |  |  |  |  |
| 1302048 | 1302048_1 | A10 | M | 155 | 2.4 | Natural | Dead |  |  |  |  | Weak body conditions, dead 0.5 day after birth |
|  | 1500526 | 17 | F |  | 4.6 | Natural | Live |  |  |  |  |  |
| 1300290 | 1500457 | 18 | M | 154 | 3.7 | Natural | Live |  |  |  |  |  |
|  | 1500427 | 19 | M |  | 3.5 | Natural | Live |  |  |  |  |  |
| 130052 | 1500534 | 20 | F | 152 | 4.6 | Natural | Live |  |  |  |  |  |
| 1100660 | 1500477 | 21 | M | 150 | 4.1 | Natural | Live |  |  |  |  |  |
| 12 | 1500471 | 22 | M | 155 | 3.1 | Natural | Live |  |  |  |  |  |
|  | 1500425 | 23 | M |  | 4.0 | Natural | Live |  |  |  |  |  |
| 20101230 | 20101230_1 | A14 | F | 150 | 2.6 | Natural | Dead |  |  |  |  | Weak body conditions, lack of milk, dead 1 day after birth |
| 110282 | 110282_1 | A11 | M | 155 | 7.0 | Stillbirth | Dead |  |  |  |  | Stillbirth by large bodies |
|  | 110282_2 |  | F |  | 2.4 | Stillbirth | Dead |  |  |  |  |  |
| 1302052 | 1500508 | 24 | F | 154 | 4.58 | Natural | Live |  |  |  |  |  |
| 090022 | 1500475 | 25 | M | 152 | 4.7 | Cesarean | Live |  |  |  |  |  |
| 100318 | 1500520 | 26 | F | 155 | 2.4 | Cesarean | Live |  |  |  |  |  |
| 1300482 | 1500512 | 27 | F | 156 | 3.7 | Natural | Live |  |  |  |  |  |
| 1100850 | 1100850_1 | A9 | __ | 151 | 3.6 | Stillbirth | Dead |  |  |  |  | Stillbirth |
| 1200246 | 1500451 | 28 | M | 151 | 3.02 | Natural | Live |  |  |  |  |  |
|  | 1500536 | 29 | F | 151 | 3.53 | Natural | Live |  |  |  |  |  |
| 1100886 | 1100886_1 | A16 | M | 152 | 3.85 | Natural | Dead |  |  |  |  | Head and neck lateral bending suffocation |
|  | 1500466 | 30 | F | 152 | 2.61 | Natural | Live |  |  |  |  |  |
| 110300 | 1500489 | 31 | M | 154 | 4.36 | Natural | Live |  |  |  |  |  |
|  | 1500419 | 32 | M | 154 | 4.34 | Natural | Live |  |  |  |  |  |
| 1200302 | 1200302_1 | A18 | M | 152 | 1.61 | Stillbirth | Dead |  |  |  |  | Stillbirth by low body weight |
| 1200072 | 1500463 | 33 | M | 155 | 5.07 | Natural | Live |  |  |  |  |  |
|  | 1500464 | 34 | F | 155 | 4.06 | Natural | Live |  |  |  |  |  |
| 110274 | 1500510 | 35 | F | 155 | 5.9 | Natural | Live |  |  |  |  |  |
| 1100518 | 1500485 | 36 | M | 155 | 6.00 | Natural | Live |  |  |  |  |  |
| 110344 | 110344_1 | A15 | M | 156 | 5.84 | Stillbirth | Dead |  |  |  |  | Stillbirth by large body weight |
|  | 110344_2 | A17 | M | 156 | 5.43 | Stillbirth | Dead |  |  |  |  |  |
| 1200012 | 1500455 | 37 | M | 156 | 3.09 | Natural | Live |  |  |  |  |  |
|  | 1500445 | 38 | M | 156 | 4.9 | Natural | Live |  |  |  |  |  |
| 1202014 | 1500503 | 39 | M | 156 | 4.27 | Natural | Live |  |  |  |  |  |
| 1100918 | 1500453 | 40 | M | 154 | 5 | Natural | Live |  |  |  |  |  |
| 090200 | 1500536 | 41 | F | 152 | 4.8 | Natural | Live |  |  |  |  |  |
| 100278 | 1500495 | 42 | M | 152 | 4.4 | Natural | Live |  |  |  |  |  |
| 10500 | 1500421 | 43 | M | 152 | 5.9 | Natural | Live |  |  |  |  |  |

The blue shadow areas indicate the occurrence of disruption at a given locus of each gene.

**Supplementary Table S5 Blood chemistry parameters of ten gene-modified Tan sheep.**

| **Blood chemistry**  **parameters** | **Cas9-medated**  (n=10; mean) | **Control**  (n=10; mean) | ***P* value** |
| --- | --- | --- | --- |
| ALT (U/L) | 17.13±1.99 | 15.79±1.31 | 0.581 |
| GOT (U/L) | 114.07±4.77 | 122.57±4.61 | 0.217 |
| ALP (U/L) | 386.60±62.36 | 484.60±39.65 | 0.201 |
| γ-GT (U/L) | 58.96±2.26 | 65.30±4.08 | 0.191 |
| TP (g/L) | 55.94±0.95 | 59.47±1.17 | 0.031 |
| Albumin (g/L) | 29.82±0.88 | 30.45±0.97 | 0.636 |
| Globulin (g/L) | 26.12±1.27 | 29.02±0.59 | 0.053 |
| TBIL (μmol/L) | 0.41±0.09 | 0.47±0.06 | 0.574 |
| DBIL (μmol/L) | 0.15±0.02 | 0.19±0.03 | 0.346 |
| IBIL (μmol/L) | 0.26±0.09 | 0.28±0.05 | 0.844 |
| CHE (U/L) | 35.80±4.32 | 47.20±5.64 | 0.126 |
| Urea (mmol/L) | 4.33±0.45 | 4.14±0.42 | 0.758 |
| Creatinine (μmol/L) | 62.82±2.01 | 67.33±2.34 | 0.160 |
| Ca (mmol/L) | 2.39±0.07 | 2.52±0.06 | 0.173 |
| P (mmol/L) | 2.28±0.21 | 2.83±0.18 | 0.059 |
| Na (mmol/L) | 143.01±1.33 | 144.60±2.90 | 0.624 |
| K (mmol/L) | 5.15±0.10 | 5.37±0.13 | 0.203 |
| Glucose (mmol/L) | 4.9±0.15 | 5.41±0.23 | 0.077 |
| Cys C (mg/L) | 0.04±0.004 | 0.03±0.006 | 0.504 |

ALT, alanine aminotransferase; GOT, glutamic-oxalacetic transaminase; ALP, alkaline phosphatase; γ-GT, γ- glutamyltranspetidase; TP, total protein; TBIL, total bilirubin; DBIL, direct bilirubin; IBIL, indirect bilirubin; CHE, cholinesterase; CysC, cystatin C.

**Supplementary Table S6** Growth parameters for mutant and control sheep.

| **Growth parameter** | **Mutant**^a^  (n=10; mean) | **Control**  (n=10; mean) | **P value** |
| --- | --- | --- | --- |
| Birth weight (kg) | 4.28±0.29 | 3.48±0.24 | 0.047 |
| BW at D30 (kg) | 9.86±0.74 | 7.64±0.75 | 0.049 |
| Weaning weight (D60) (kg) | 15.67±1.33 | 12.72±1.00 | 0.093 |
| BW at D90 (kg) | 20.60±1.44 | 17.51±0.93 | 0.088 |
| BW at D120 (kg) | 25.26±1.94 | 21.63±0.71 | 0.103 |
| BW at D150 (kg) | 29.19±1.97 | 24.90±0.82 | 0.047 |
| BW at D180 (kg) | 34.42±2.37 | 26.43±0.86 | 0.004 |
| BW at D210 (kg) | 38.76±2.73 | 30.53±0.80 | 0.004 |
| BW at D240 (kg) | 44.35±3.14 | 34.03±1.14 | 0.001 |
| ADG (g) (0-240 d) | 166.95±12.83 | 127.31±4.88 | 0.001 |

BW, Body weight. ADG, Average daily weight gain. ^a^In both mutant and control groups, 10 animals (six males and four females) were used for student t’ test.

**Supplementary Table S7**  Oligonucleotides for generating sgRNA expression vectors.

| MSTN sgRNA-1 top strand | tagGTCTCAGATATATCCACAGT |
| --- | --- |
| MSTN sgRNA-1 bottom strand | aaacACTGTGGATATATCTGAGA |
| MSTN sgRNA-2 top strand | TAGGATTTTGAAGCTTTTGGAT |
| MSTN sgRNA-2 bottom strand | aaacATCCAAAAGCTTCAAAAT |
| ASIP sgRNA-1 top strand | ccggCTTCAGGTTCCTTTCATCTC |
| ASIP sgRNA-1 bottom strand | aaacGAGATGAAAGGAACCTGAAG |
| ASIP sgRNA-2 top strand | CCGGCAATTCTTCCATGAACCTGT |
| ASIP sgRNA-2 bottom strand | aaacACAGGTTCATGGAAGAATTG |
| BCO2 sgRNA-1 top strand | ccgGTTAGAAGCGGTGCAATGCA |
| BCO2 sgRNA-1 bottom strand | aaacTGCATTGCACCGCTTCTAA |
| BCO2 sgRNA-2 top strand | ccGGTCGTCTCAGCTCGAGTCC |
| BCO2 sgRNA-2 bottom strand | aaacGGACTCGAGCTGAGACGA |

**Supplementary Table S8** Primers for genotyping and amplifying Cas9/sgRNA targeted *MSTN*, *ASIP* and *BCO2* fragment.

| **Gene** | **Name of primer** | **Sequence** | **Amplicon (bp)** |
| --- | --- | --- | --- |
| *MSTN* | MSTN E2 Forward | GACATGGAGGCGTTCGTTCATT | 422 |
|  | MSTN E2 Reverse | CTGGGAAGGTTACAGCAAGATCA |  |
|  | MSTN E3 Forward | TAGAAGTCAAGGTAACAGACAC | 509 |
|  | MSTN E3 Reverse | GTTCATATACTGTAGCTTGTGC |  |
| *ASIP* | sASIP CKE1 1F | GTTCTCCTTCCATGTCCTAAGC | 541 |
|  | sASIP CKE1 1R | ACTAGATTGGTGAGAGGCAGAG |  |
| *BCO2* | sBCO2 CK 1F | ATTGCGGTTCACAAGTGGAGAA | 500 |
|  | sBCO2 CK 1R | AGGGATTGTGTAGCAATGTTAGC |  |
